# Re-written narrative: transformation of the image of Ivan-chaj in Eastern Europe

**DOI:** 10.1016/j.heliyon.2020.e04632

**Published:** 2020-08-19

**Authors:** Julia Prakofjewa, Raivo Kalle, Olga Belichenko, Valeria Kolosova, Renata Sõukand

**Affiliations:** a Ca’ Foscari University of Venice, Italy; b University of Gastronomic Sciences of Pollenzo, Italy; c Institute for Linguistic Studies, Russian Academy of Sciences, Russia

**Keywords:** Social media, Viral narrative, Youtube, Twitter, Instagram, Memes, *Epilobium angustifolium*, Sociology, Information science, Mass media, Knowledge representation

## Abstract

The aim of this study was to understand the role of viral narratives and the involvement of social media into the invention of tradition. We took as an example the recently highly promoted Ivan-chaj, a tea made from the fermented leaves of willowherb, a plant little known and used in Europe until a few years ago. Relying on a wide variety of sources circulating on the Internet (videos, various texts and visuals) and robust empirical field research results, we used mixed methods to analyze this specific case in order to understand if people adopt new teachings and if their acceptance leads to practical output. The results showed that the new teachings spread quickly, supported by narratives based on a wide variety of interaction points that viralized the message, also causing an economic impact. It is clear that the change of status and the economic success that Ivan-chaj now enjoys is due to the virality of the narrative, which has reshaped the image of Ivan-chaj from an “outcast” imitation and tea substitute into the national healthy drink. Having appeared in Russia, mostly as a Russian cultural marker, the narrative went viral and spread beyond its borders where neighbors have tried in turn to embrace Ivan-chaj as their own cultural marker by proclaiming it a local tradition. Indeed, narratives regarding Ivan-chaj spread easily in countries sharing some linguistic, historical and/or cultural elements with Russia (via the nexus of the Soviet Union).

## 1 Introduction

The Internet and social media provide a powerful avenue for information exchange and the development of opinions on a diversity of issues. The wide variety of sources makes it difficult for a reader to navigate among different trustworthy or/and misleading stories, suggestions and recommendations freely accessible through social media, often amplifying peoples' fears and hopes. Many of the subjects involved in viral stories existed in pre-internet times. For example, the anti-vaccination movement started long before the widespread use of the Internet (see Gangarosa et al., 1998 for analysis of its impact), so the later intensive anti-vax attack of Russian troll farms (Broniatowski et al., 2018) sowed the seed of distrust on fertile ground. As with the anti-vax movement, the influencing of the 2006 Israeli election through fake political news (Balmas, 2012) reflects the effects of fake news on potentially life-changing decisions. Yet, misleading stories can provoke not only decisions, but also practices, and therefore there is a need for an interdisciplinary approach in order to reduce their spread and “to address the underlying pathologies it has revealed” (Lazer et al., 2018). There is a growing body of research on the mechanisms of the spread of information and methods to analyze the information itself (Colliander, 2019; Wang et al., 2019; Introne et al., 2018; Vosoughi et al., 2018 and references therein), yet more research is still needed. To understand if and how social media can help to invent tradition (sensu Hobsbawm and Ranger, 1983) we need to analyze something not noticed earlier that has recently become popular.

A good candidate for such an exploration is Ivan-chaj, a beverage (hereafter referred to as Ivan-chaj) prepared from the fermented leaves of rosebay willowherb *Epilobium angustifolium* L. (hereafter referred to as willowherb). It is important to note that Ivan-chaj is also the name of the plant in Russian, along with other names like kiprej and koporskij chaj, but we will use this term only to refer to the beverage. Making tea is very practical work, demanding several skills. One needs to be able to recognize the plant, and know its habitat, time of collection, method of collection, processing and preservation techniques, etc. While in the last six to seven years, sporadic use of dried (not fermented) leaves and flowers of willowherb for making tea has been documented in some Slavic-speaking regions (e.g. Sõukand et al., 2013; Stryamets et al., 2015; Sõukand and Pieroni, 2016; Sõukand et al., 2017; Pieroni and Sõukand, 2018), the people mentioning their use have been very few. Fieldwork carried out in summer 2018, however, showed a comparably wide use of Ivan-chaj in several of the researched sites, especially in the Russian Federation, where around a quarter of the interviewees reported the current use of Ivan-chaj as a recreational tea (Kalle et al., 2020). Significantly, Kalle et al. (2020) demonstrated that while the specifically processed leaves of willowherb were initially sold as fake black tea in the 18th century and later sporadically promoted and used as a tea substitute in the European part of the current Russian Federation, the fermented version of Ivan-chaj have been used mainly within the last five years.

A basic Internet search revealed that social media is overflowing with information on Ivan-chaj and its “glorious” history which uses elements of the past in order to build a believable narrative. Most of them were published in the last six to seven years and have presented Ivan-chaj as a drink that has been used in Russia for centuries (Voronina, 2010; Sychyova et al., 2016; and many others). Yet, the “history” represented on social media is far from reality, forming *in corpore* a perfect example of a false narrative (*sensu* Introne et al., 2018). The rapid rise of interest in Ivan-chaj has happened within the last decade, which coincides with the expansion of the social network itself; however, it is not solely a social media-based phenomenon.

The aim of this study was to understand the role of fake news and the involvement of social media in the construction of the symbolic identity narrative and, through that, the invention of “tradition”. To this end, we 1) recorded and analyzed current practices in selected regions potentially affected by social media, 2) recorded and analyzed current reflections on Ivan-chaj on various social media, and 3) evaluated the mechanisms supporting the current popularity of Ivan-chaj.

## 2 Data and methods

Our data consist of a variety of sources: videos, texts and visuals circulating on the Internet, and robust empirical field material collected through face-to-face conversations, without involvement of the Internet. The complexity of the subject required the use of mixed methods for analysis.

### 2.1 Field study and data analysis

In order to understand if and how Internet-based narratives affect practice, we relied on qualitative analysis of the responses of 240 people interviewed in summer 2018 in three regions: the Republic of Karelia, Russian Federation (37 of the 70 people interviewed mentioned Ivan-chaj), Setumaa and Võrumaa in Estonia (16/95), and the border region between Lithuania and Belarus (6/141). The proportion of the people that mentioned the use of Ivan-chaj was higher in the region closer to the “historical center” of the narrative, decreasing with distance. The general framework of the interviews, which lasted from 30 min to 3 h, was about personal experience using wild food and medicinal plants in each specific region. In each location, we interviewed the major linguistic and/or ethnic groups: Karelians, Russians and a few newcomers from other parts of the former Soviet Union in the Russian Federation; Setu, Estonians and Russians in Estonia; and Lithuanians and Poles in the Lithuanian-Belarusian borderland. We used pseudo-random and snowball methods to identify the interview subjects. As this was a part of a larger cross-border ethnobotanical research project investigating the effect of different socio-political scenarios on Local Ecological Practice, Ivan-chaj was named among many other food and medicinal plants. The initial information on the use of willowherb was mainly provided spontaneously, with brief ad hoc questions asked afterward. The structure of the questionnaire was built on the basis of dishes and diseases, so the plant was rarely asked about explicitly. There were two exceptional cases in which the interviewer did explicitly ask if the participant used Ivan-chaj, as their neighbors suggested that they did – the answer, in both cases, was negative.

The study was approved by the Ethics Committee of Università Ca' Foscari and strictly followed the ethical guidelines outlined by the International Society of Ethnobiology (ISE, 2008).

The interview transcripts were entered into RQDA software (Huang, 2010) and a keyword list was developed on the basis of the content analysis of subjects that arose during the interviews. The emic keywords from the transcribed interviews were grouped according to their similarity in meaning, using the exact wording of interviewees as much as possible. The subsequent analysis was guided by the logic of the results.

### 2.2 Media as a resource

For understanding the representation of Ivan-chaj in the media we extracted several datasets from online sources like Google trends, websites (e.g. blogs, micro-blogs, and forums), and social networks (e.g. Twitter, YouTube, and Instagram) (Table 1, Supplement 1). These platforms are quite popular channels in the study area for sharing and accessing media, including pictures, videos, and texts.

**Table 1:** The various media sources analyzed.

What is analyzed	Source	Number	Time of extraction	Type of analysis
Sample analysis				

Narratives	blogs, homepages, forums	370	Oct-18	timeline, content
Memes		17		content

Systematic analysis				

Tweets	Twitter	446	Oct-19	timeline, content
Photos	Instagram	1713		
Videos	YouTube	513		
**Total sum**		3062		

We used only publicly available data (users with privacy restrictions were not included in our dataset) by using the API (application programming interface). The data for the sample analysis was collected using snowball and subject saturation methods, while for extracting data for systematic analysis R software (Version 3.6.1) was used (Danneman and Heimann, 2014). After data scraping we conducted data cleaning, deleted duplicates, removed spaces and non-printing characters, transformed and rearranged columns, fixed dates, etc.

A total of 3062 samples, posted by active users, were accessed. The content was manually classified and double-checked for consistencies. We stored a variety of metadata for each of these posts. The most important of which were data about social activity (“likes”, “dislikes”, “shares” and comments).

The keywords used to detect the data in the different Internet environments and online social media were *“иван-чай”* and its Latinized equivalent *“ivanchai”* for all but written narratives, for which the searches were conducted in the languages of the areas under investigation and their immediate surrounding territories: Russian *“иван-чай”, “копорский чай”*; Belarusian *“іван-чай”, “скрыпень”*; Ukrainian *“скрипень”*; Lithuanian *“gaurometis”, “ivan arbata”, “ivan čaj”*; Latvian *“šaurlapu ugunspuku”*; Estonian *“ivanatee”, “ivan tsai”, “ivan(i) tee”, “põdrakanepi tee”*; Finnish *“maitohorsma”, “Ivan ceai”*; Polish *“iwan czaj, “wierzbówka kiprzyca”*; and Romanian “*ceaiul lui Ivan*”.

For making timelines, we used Excel and RAW Graphs, while for the content analysis we used different approaches depending on the type of media. For YouTube and Instagram we identified the most used words presented in the title of the video or description of the photo, respectively. The visuals were chosen for analysis on the basis of diversity, in an attempt to form a thematic overview of the parodies. The written narratives were thoroughly studied and qualitatively categorized in different plots according to the ad hoc sub-themes covered. We applied qualitative content analysis to explore the narrative structure. Frequently appearing words with regard to plots about drinks and plants were visualized using word clouds.

## 3 Results and discussion

### 3.1 Ivan-chaj in practice

#### 3.1.1 Republic of Karelia

The high percentage of interviewees from the Republic of Karelia (Russian Federation) familiar with Ivan-chaj is not surprising as the region is quite close to the claimed place of origin of Ivan-chaj production (Koporye, Leningrad Oblast). Nevertheless, the subjects addressed by those people in relation to Ivan-chaj are diverse and the history of use not straightforward. A Karelian woman born in 1955 narrated: “*Now Ivan-chaj is widely sold. It is fashionable now. I once tasted it but did not like it; it is necessary to do it the right way, to catch the phase, and to have a dryer.*” Only one Russian woman (b. 1966) claimed to have made Ivan-chaj throughout her life: “*We have always had herbal teas. Even now I drink koporskij chaj more often than usual tea. I make it myself, I ferment it. It becomes very fragrant, pure honey. [I use] only leaves. If I want – drink it pure and clean, if I want – add some currants.*”

##### 3.1.1.1 Current use

**Figure 1:**
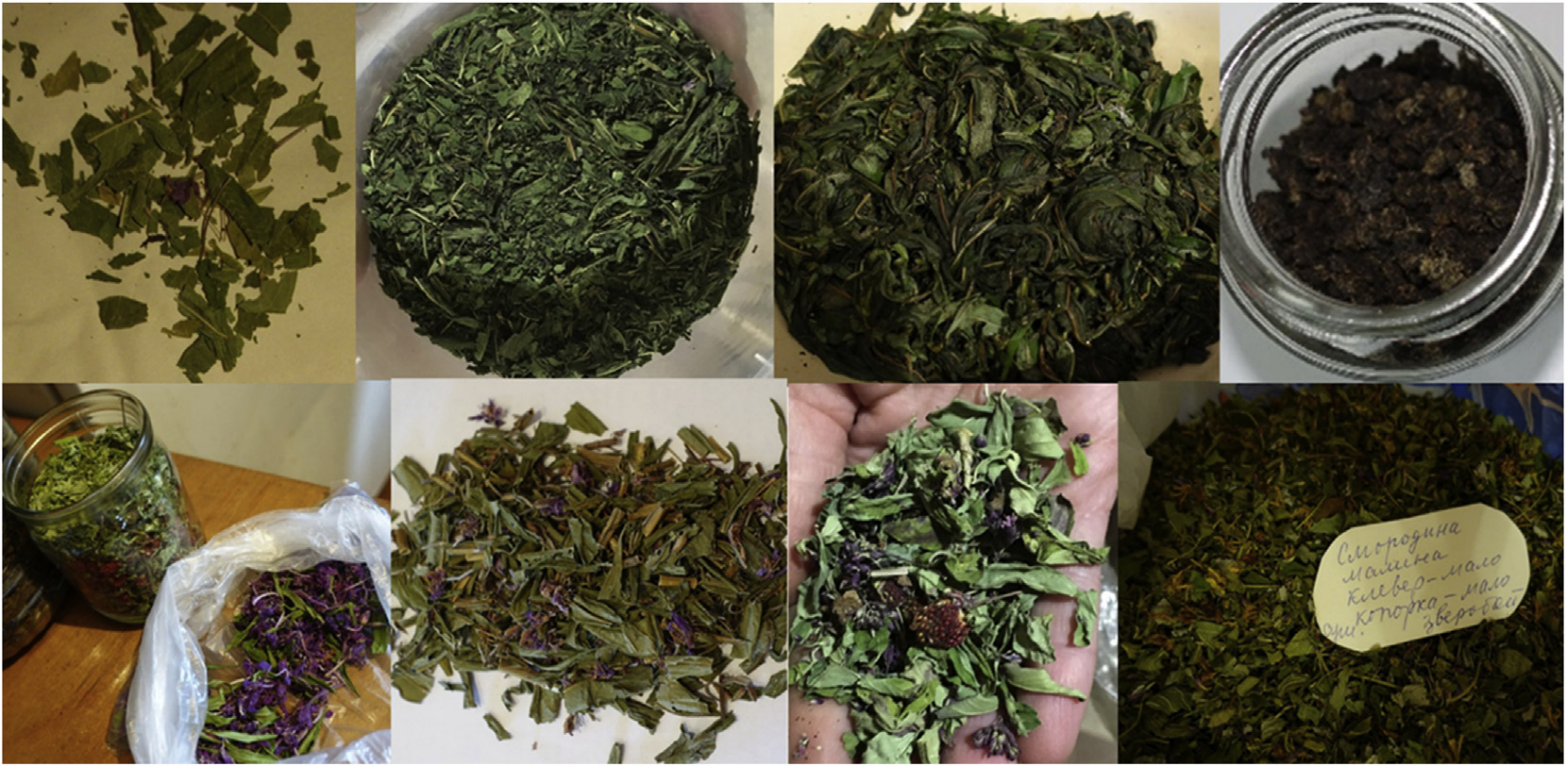
Examples of various homemade Ivan-chaj preparations produced in the Republic of Karelia by interviewees for their own consumption. Upper row: different stages of the fermentation of leaves; lower row: mixtures of dried leaves, flowers and other plants. Photos by Valeria Kolosova and Olga Belichenko 2018.

The content regarding current use is often controversial. Many of our interviewees had fermented or dried Ivan-chaj at home, either done themselves, bought or received as a present (Figure 1). Ten people claimed to make Ivan-chaj themselves, and eight described the technology of fermentation (each with different wording). Three people also described experimenting after seeing teachings on the Internet and two people claimed to have learned from others. Five people described the technology as complicated. Eight people asserted that others make Ivan-chaj and three of them suggested that it has now become a fashionable thing. One man (b. 1946) said that he wants to try it, but had not yet had the chance: “*I wanted Ivan-chaj, it is promoted on TV, but I have not yet tried it. On TV they told us that in Russia Ivan-chaj was drunk before; I don't remember what broadcast it was; it was about the tea, various countries and that now we had forgotten Ivan-chaj*”. One Russian woman, born in 1978, who makes rag dolls and teaches this in workshops, learned from medicinal books that willowherb was poisonous: “*I drank Ivan-chaj until I realized that I have [health] problems because of it. I dry it simply for the dolls. It goes into dolls as stuffing material*”. A woman of Belarusian origin, born in 1955, narrated: “*As the Chinese say, if Ivan-chaj were to grow in China, every Chinese would be a millionaire. Here it grows, but we don't really use it...*” A Russian woman, born in 1986, who produces Ivan-chaj narrated:

“A few years ago, when we started making tea, we had several blends. And only we made complex blends with black tea. That is, people made all kinds of herbs, mixed them, but it wasn't with black tea. And we were thinking - why not smoke it? And somehow we smoked, and so far no one else does it. We first ferment it. There were classic recipes on the Internet, there are one million one hundred thousand of them, but in the end we developed our own. Now you can see it - it will be yellow or red, it is not yet ready. Fermentation has not yet been completed, but in winter it will be just right, it needs to stay for another six months. When we realized that our product was starting to be in demand, we realized that this could generate income. We are constantly studying the market in order to survive, because a small Ivan-chaj industry has appeared in Karelia and this is sad for all manufacturers. Not only in terms of income, but in terms of quality.”

Those who indicated the start of the practice spontaneously mentioned one to four years ago. There was one exception: a Russian woman born in 1935 mentioned 1989 as the starting year (a time when various recipes of fermented Ivan-chaj were published; Koshheev, 1989): “*No, it wasn't done here earlier. I think Ivan-chaj is used now particularly a lot, I know that for sure. And they always ferment it, someone came and taught us in 1989. From then Ivan-chaj has been used until now*”.

##### 3.1.1.2 Past uses

Eleven people claimed to have used willowherb tea in the past, but only four of them stated that they personally drank it in their childhood as a tea, while five asserted that past use was restricted to flowers. One Karelian woman (b. 1972) told us that her mother recalled drying Ivan-chaj, while another woman born in 1966 mentioned that her grandmother considered it a famine food that she never used afterward. Four people claimed that they think that it may have been used in the past. At the same time, three people asserted that their family buys black tea, seven people were convinced that Ivan-chaj was not used in their childhood, and one person recalled that only a few individuals used Ivan-chaj in the past. Three people described its past use for animal fodder: “*willowherb was collected in childhood to sell to sovkhoz as food for polar foxes and minks. All children of my generation did it. We did not drink it ourselves*” (Karelian woman, b. 1954).

#### 3.1.2 Outside the Russian Federation

In Estonia, a woman with higher education (b. 1954) referred directly to pseudo-history: “*Ivan-tšai's history is such that in Russia there was a factory where Ivan-chaj was made. The first thing that the Germans destroyed was the factory, because then the Russian soldiers had nowhere from which to get their strength”.* As she was selling medicinal plants, she also copied teachings from the Internet, which she gave with the tea pack. Another Estonian woman (b. 1948) created her own story: “*Now it is called ivan-tšai, this name came from Russia. I always say to the men that this is the plant that Peter I always gave his soldiers as a compulsory drink because it takes care of men's potency. He was the first to call it ivan-tšai*”. However, on the Estonian-language Internet, there is only a single statement that Peter I was the first to begin drinking black Chinese tea in Russia. In addition, two people knew that this was the drink of wealthy men in the past, stating: “*I read that the Russian boyar had used this tea and that it was also traded. It was very profitable in earlier times but I do not know where it was sold ...”* (woman, b. 1957). Also, “*Russians make the tea, call it ivan-tšai or tsaar-tšai. I've picked it myself; it should be good for men, for the prostate*” (woman, b. 1938).

In Lithuania, narratives revealed the very recent origin of the practice and the described confusion introduced through social media. A Polish-speaking woman (b. 1941) referred to *Hypericum perforatum* L. as “Ivan-chaj”. She associated the Russian name of Ivan with the Catholic Saint John and said: “*Świętojańskie ziele - it is in our language. And easier for you* [to understand] *- Ivan-chai*”. The most enthusiastic users of Ivan-chaj in the study region were an Old Believer (b. 1968) and his wife of Polish origin (b. 1972). They collected flowering tops of willowherb, having learned about this plant on the Internet. They were waiting for the start of winter to drink it. The couple remarked that local Polish and Lithuanian people are not familiar with the tradition of using Ivan-chaj. They experimented with tea fermentation: the husband thinks that longer fermentation yields a greater taste, but his wife said that longer fermentation kills the aroma. As a compromise, they fermented willowherb for 4 days. A man of mixed Lithuanian-Polish ancestry (b. 1963) also experimented with tea fermentation. He made different teas from willowherb by altering the fermentation and drying process. He knows many recipes from books and the Internet and tries to choose the best ones.

The difference in manufacturing technology is the clear marker identifying current uses prompted by social media. The way to ferment the leaves of willowherb into proper Ivan-chaj is mainly learned from the Internet or through mediators, often experimenting with different suggested recipes. People re-narrate stories found on the Internet or other media, which they are eager to alter according to their understanding and preferences, often taking earlier use for granted and stories being true without questioning their provenance.

### 3.2 Ivan-chaj story in the media

#### 3.2.1 The start of the campaign

**Figure 2:**
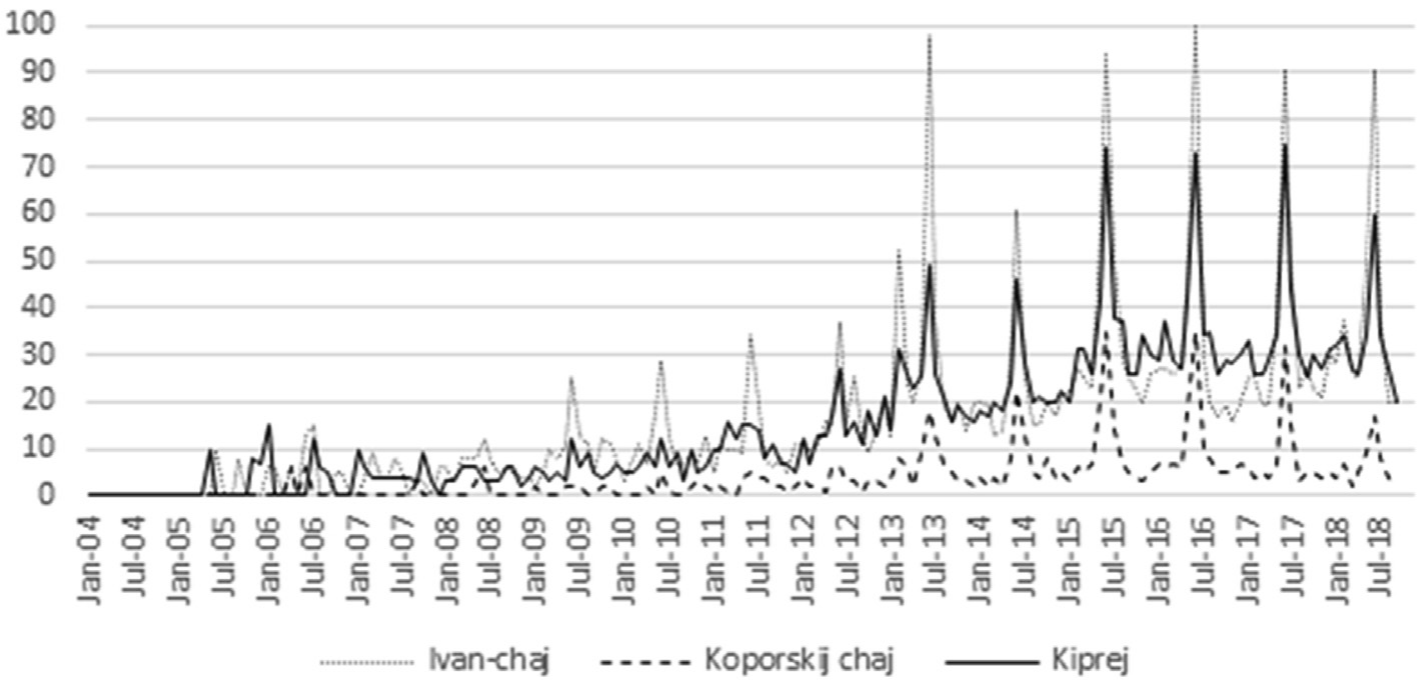
Google trends for proportional global interest in Ivan-chaj and its other two most widespread names (source: trends.google.com). January 2004–October 2018.

Google trends showed a sudden global increase in the interest in Ivan-chaj starting July 2013. The search rate increase repeats itself yearly and accelerates during the summer months with a peak in July when the plant is collected (Figure 2).

Television might have given the first push: in March 2009 tea made of willowherb was mentioned on the program *Dobroye utro, Rossiya!* [Good morning, Russia] on the Rossiya-2 TV channel (Russkij chaj, 2009).

The first great rise in interest coincided with the date (March 2013) when the Russian channel RenTV aired a TV program dedicated to Ivan-chaj (Pishcha bogov, 2013). This channel, blacklisted for spreading fake news, colorfully illustrated its “glorious history”.

##### 3.2.1.1 Pseudo-history narratives

**Figure 3:**
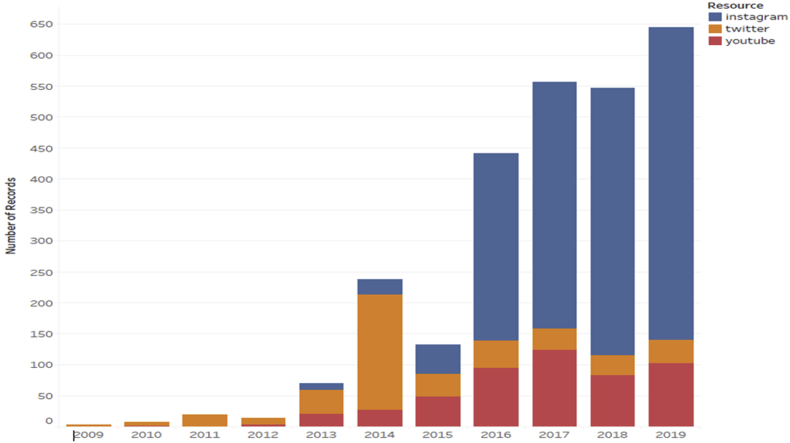
Timelines of publishing Ivan-chaj related content on twitter.com, youtube.com and instagram.com.

We found 160 original narrative segments representing all languages except for Finnish. Analyzing the content of the narrative lines (Figure 3), we divided the resulting 43 plots into two separate categories: narratives about the **plant** and narratives about the **drink**. The narratives about the plant (willowherb) cover its origin and properties of the plant and the origin of the name. The narratives about Ivan-chaj as a fermented drink cover its origin, knowledge, medicinal properties, relation to identity and economic importance. None of the narratives have an actual historical basis and the diversity of the covered subjects is impressively wide.

A large number of storylines refer to historical figures and events (not necessary factual) familiar in the specific region which they are addressing, building on the audience's existing knowledge. It is clearly evident that the Russian idea of cultural identity established through Ivan-chaj was mirrored in other countries, local origin of the tradition of drinking Ivan-chaj. If in one case there was a reference to common roots or it referred to Slavic (Russian) unity, there were several cases pinpointing a specific ethnic origin (Finno-Ugric, Baltic, Belarusian, Kievan Rus'). A narrative line of ancestral roots (found only outside Russia) highlights the construction of cultural identity and strategies of ethnic survival through Ivan-chaj.

**Figure 4:**
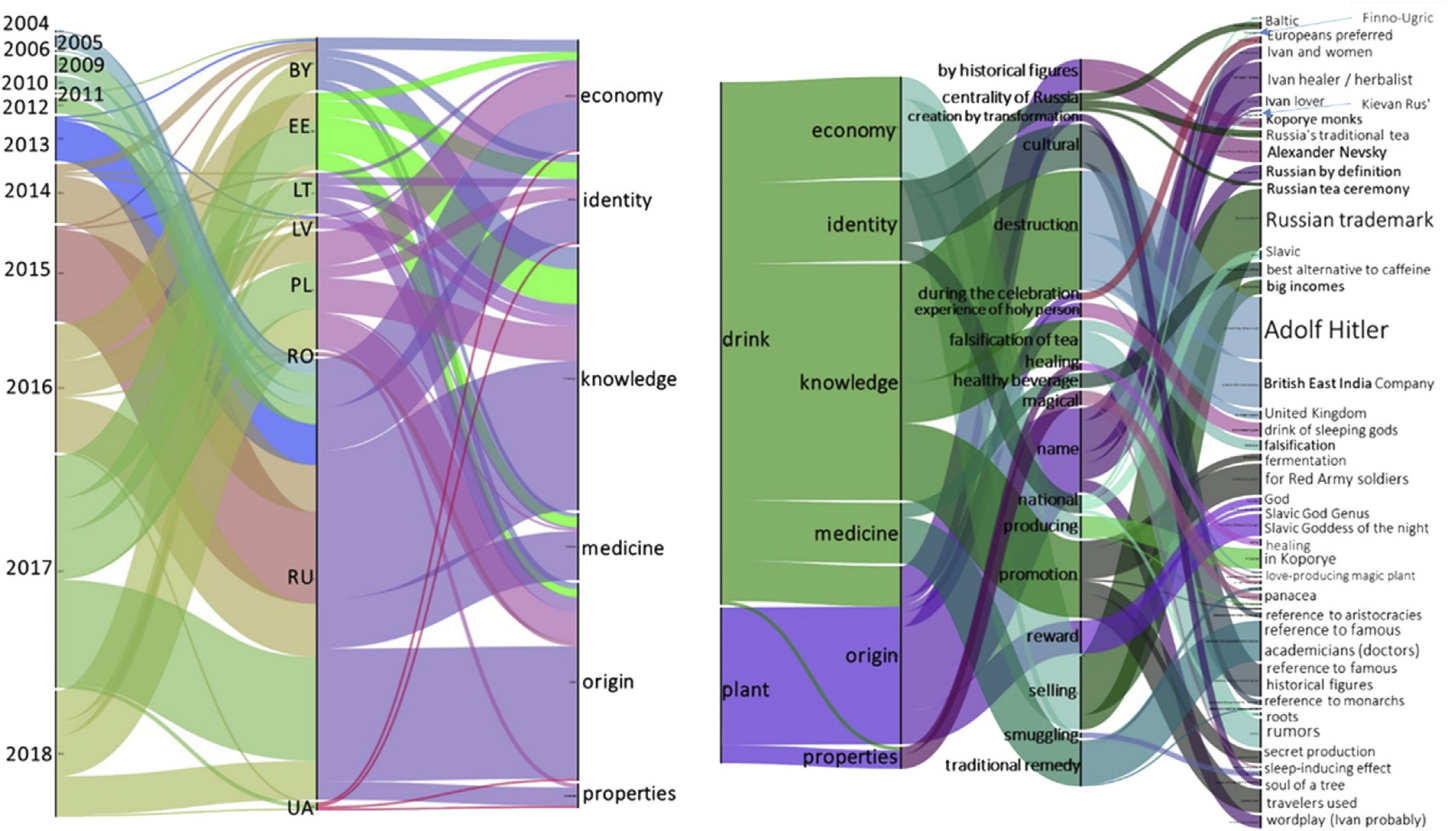
Alluvial diagram of Ivan-chaj narratives by language, year of publication and subjects covered based on 370 narratives.

As shown in Figure 4, the narratives originating from Russia clearly dominate the data and were produced in all years and cover all subjects. Some narratives did not leave the borders of Russia, while many others are spread across almost all studied languages and a small proportion is dispersed in specific countries. Remarkably, until 2013 no narrative was translated into other languages and only since 2016 have the pseudo-stories gone viral across borders and moved from the Russian-speaking center to many of the countries covered in this study through translation into national languages. The two narratives encountered in Romanian are related only to the origin of the plant name, while the modest representation of narratives in Latvian, Belarusian and Ukrainian may be due to the Russian language still serving as the *lingua franca* in those countries. We might consider this broadening of narrative frames through which Ivan-chai tea is reported as reflecting what Gurevitch et al. (1991) refer to as “domestication”, since a key feature of the domestication process is the way in which globally significant events, and here we would include Ivan-chaj, are rendered “comprehensible, appealing and “relevant” through reference to a “narrative framework that is already familiar to and recognizable by” domestic audiences (Gurevitch et al., 1991: 206–7).

Finally, we have an image of Ivan-chaj, spreading on blogs, micro-blogs and local forums, as a healthy, trendy, and delicious high-class beverage for high-class people. The studied narratives have very detailed storylines, which are typical for newly created ones (Radchenko, 2016). There is also a strong folklorization (Laferté, 2003) of the plant and the drink in contemporary discourse, and the mythologization of this phenomenon (Sola Morales, 2013). We can also trace the adoration and exclusivity of this drink, as well as the creation and dissemination of new drinking rituals (for example, Ivan-chaj drinking ceremonies). The ritualization of consumption through social media exposure adds to what Grasseni called the virtualization of local foods (Grasseni, 2007). As a result, in social media, TV programs, festivals and advertisements, Ivan-chaj is ever more depicted as a symbol of specific territories, through reference to national and cultural identity. It is consequently treated as a political and economic resource. To claim “local” status for a drink not only means re-inventing (just as “traditions” are invented or constructed): it can mobilize strategies of self-rediscovery of the patrimonialization of local histories, places and landscapes.

#### 3.2.2 The potential impact of social media

Social media is highly visual: images can be sent and received as messages (Nakamura, 2008), occupying a key place in communication and information sharing on online platforms. Ivan-chaj has inspired an impressive number of images, tweets and videos (Table 2). The emergence of social media is providing an alternative avenue for information exchange and opinion formation on related issues. Collective online discourse in such media leads to the formation of a complex narrative - reaction, conveying public views and perceptions. Ultimately, we argue that collected data has potential value in helping us understand the social experience of the practical uses of Ivan-chaj, but studying these types of data presents theoretical and methodological challenges (Murthy and Gross, 2017).

**Table 2:** Impact of selected social media channels in spreading information on Ivan-chaj.

Characteristics	Instagram	Twitter	YouTube
Number of reposts	-	2871	-
Number of creators	676	398	421
Maximum number of posts per creator	141	133	22
Proportion of creators with one post	31%	49%	60%
Number of likes	102225	5257	41395
Number of dislikes	-	-	2718
Number of comments	-	312	-
Number of views	-	-	15807281

**Figure 5:**
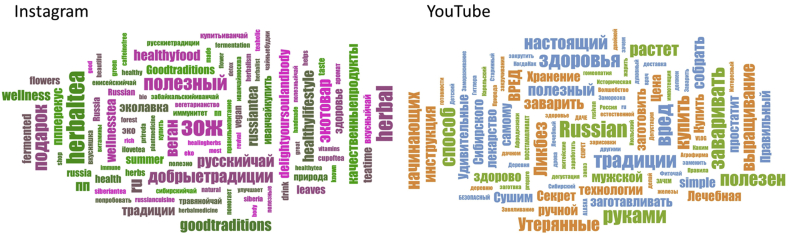
Word clouds of the most often repeated words in descriptions of Instagram pictures and titles of YouTube videos.

As depicted in Figure 5, “ЗОЖ (здоровый образ жизни)” (“healthy lifestyle”) and “Russian” were the most frequent words in the Instagram and Youtube datasets. Among users of Instagram, **health-related** terms such as “herbaltea,” “healthyfood”, “health” “healingherbs”, “полезный” (“helpful”), “ппперекус” (“proper nutrition snack”), “plantmedicine” and “wellness” were observed, indicating that users mostly shared information about the health properties of the studied plant. We also found identity-specific words such as “russiantea”, “Russia”, “russiancuisine”, “ru”, and “сибирский чай” (“Siberian tea”). Among users of Youtube, **practice-related** terms such as “готовим” (“cook”), “сбор (собрать)” (“collecting (collect)”), “сушить” (“dry”), “выращивание” (“growing”), “произодство” (“producing”) were frequently noted, indicating that these were important words for characterizing new practices. Also, frequently encountered terms in the Youtube data corpus about life hacks (“правильно” (“right”), “настоящий (“real”) “секрет”, (“secret”)) are about how to better collect the plant and prepare the drink.

Another important trend is that 72% of the videos were posted by men, which is not characteristic of traditional knowledge transfer mechanisms in Slavic countries, where the domain of plants is dominated by women.

#### 3.2.3 Jokes as a statement

Collective discourse in online social media leads to the formation of a complex narrative, conveying public views and perceptions. Some humorous posts start ‘trending’, becoming the most circulated (Murthy and Gross, 2017). In a digital economy where attention is scarce, images are a quick and efficient way to communicate thoughts and feelings (Pittman and Reich, 2016). We found that the production and consumption of humorous texts and images highlighted changes in the representation of the studied plant – a potential turn from top-down understanding of the plant to bottom-up, citizen informed views. While many such images contain references to Russia dominance, they also ridicule this new symbol: the theme of powerful and healthy beverages in these images and texts provide a counter-narrative to mainstream media accounts.

**Figure 6:**
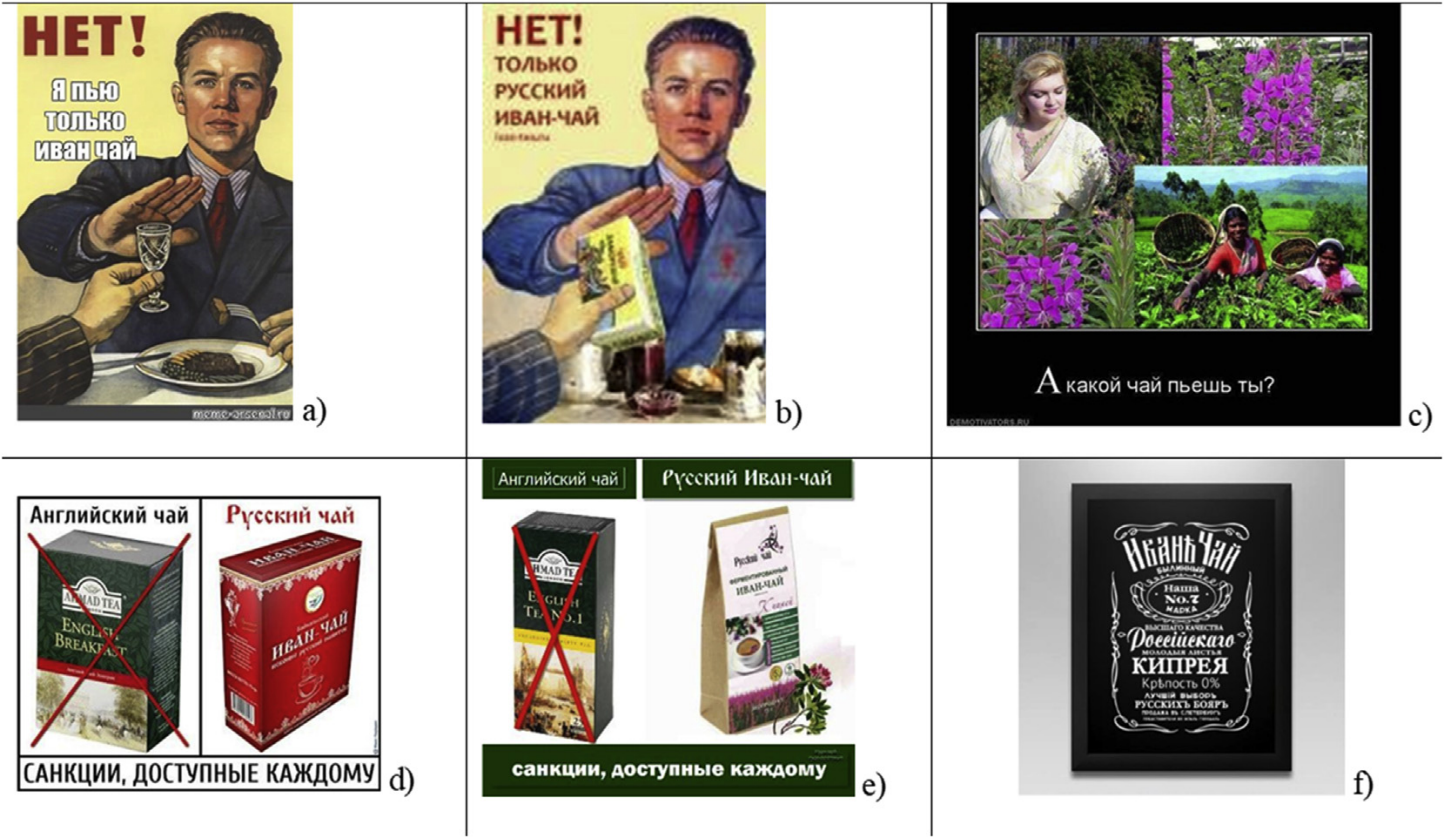
a) *“No! I drink only Ivan tea”* (meme-arsenal.ru); b) *“No! Only Russian Ivan-tea”* (vk.com); c) *“And which tea do you drink?”* (demoviators.ru); d) *“English tea vs Russian tea. Sanctions made available to everyone”* (vk.com); e) *“English tea vs Russian Ivan tea. Sanctions made available to everyone”* (vk.com); f) *“Ivan tea. Epic. Our brand. Russian highest quality. Young leaves of fireweed. Alcohol 0%. The best choice of Russian boyars. Sale in St. Petersburg. Representatives in all cities”* (vk.com).

The range of humorous reactions covering the subject of Ivan-chaj can be divided into five groups. The first group can be labeled as *“Drink Russian!”* (Figure 6). For instance, one of the memes referred to one of the most changed Soviet propaganda posters combating alcoholism – “Нет!” (“No!”). In the modern context, one of the memetic replacements takes place with Ivan-chaj. Accommodation via references to local culture creates a feeling of recognition and commonality and renders the process of entextualization smoother. One of the vernacular reactions to international sanctions against Russia over the Ukraine crisis was memes that offered Ivan-chaj as an alternative to importing English tea.

Memes and texts circulating on Twitter about Ivan-chaj are mostly stereotypical: *“- Здравствуйте, сударь! - Мне блин с картошкой и салат “русский цезарь”. - Пить что будете? - Иван-чай. - Сударь, не хотите ли к заказу еще взять брошюрку о спасителе белой расы Адольфе Гитлере? - Что? - Что?*” (“- Hello, sir! – For me, please, pancake with potatoes and “Russian Caesar” salad. - What will you drink? – Ivan-chaj. - Sir, would you like to order a booklet about the savior of the white race Adolf Hitler? - What? - What?”) (Twitter, user @Zh-d, 30-Aug-19). Ivan-chaj equally became an object of ridicule. Many tweets consider drinks as the main actor in life: “*Мужчины в моей жизни: - Max Factor, - Дымов. – Рентген. - Mr. Proper. - Б.Ю. Александров. - Иван-чай. Ни о чом не жалею*” (“Men in my life: - Max Factor, - Dymov [*brand of sausages and meat delicacies*] - Röntgen. - Mr. Proper. - B.Yu. Alexandrov [*chocolate glazed curd cheese brand*]. – Ivan-chaj. I have no regrets”) (Twitter, user @n-a, 1-Sep-19).

In contrast to the “drink national beverages” narrative, the joke is a reference to the label of the top-selling American whiskey Jack Daniel's, converted into Ivan-chaj. Or on Twitter “*срочная новость. кока-колу переименовали в иван-чай*” (“Breaking news. Coca-Cola was renamed Ivan-chaj”) (Twitter, user @ni-os, 13-May-19). *“- Что это? - Иван чай. - А можно мне чай без Ивана?” ("- What is it? – Ivan-chaj. “Can I take tea without Ivan?”)* (Twitter, user @P-4, 12-Jun-16).

**Figure 7:**
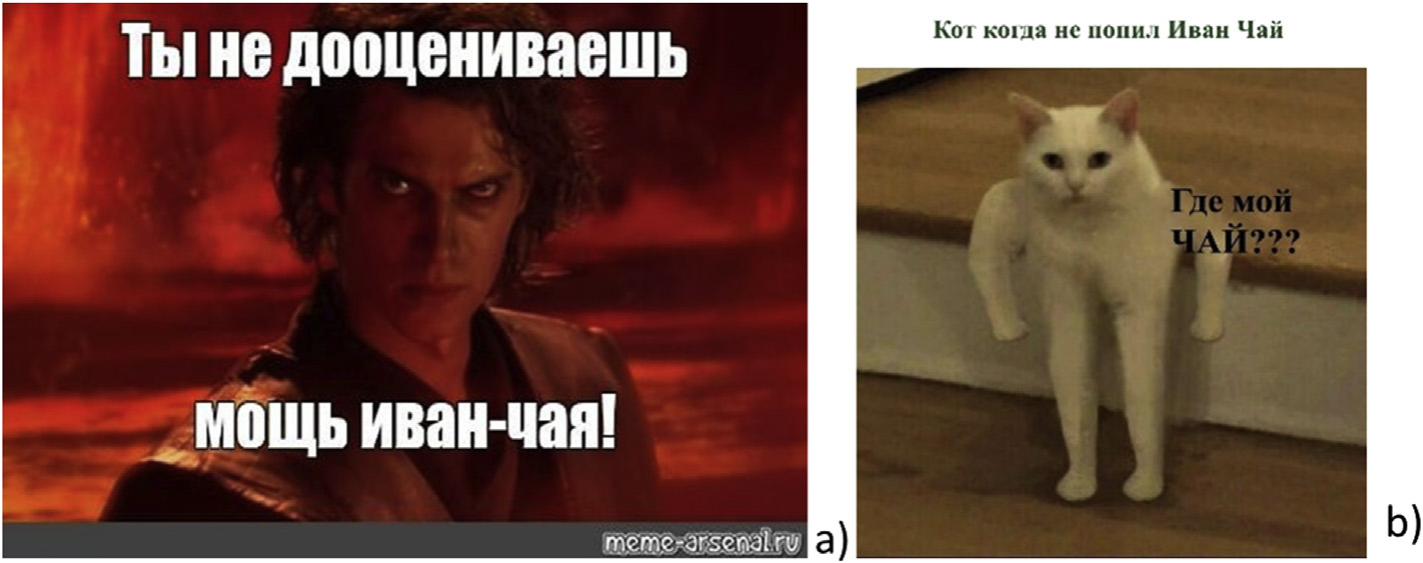
a) *“You underestimate the power of Ivan tea!”* (meme-arsenal.ru) b) *“When the cat didn't drink Ivan tea. - Where is my TEA?”* (vk.com).

Another group of humorous reactions (Figure 7) refers to a value set that includes the power of Ivan-chaj (a reference to Star Wars Episode III) and it's magical health.

In contrast, the next group of Ivan tea jokes references delirium tremens after drinking/smoking Ivan-tea (popular Soviet film “Kidnapping, Caucasian Style”: “*We will cure you - alcoholics are our profile*”). In the next case, the authors of the visual narratives emphasize that it is not so easy to abandon the use of Ivan tea (“*One does not simply walk into Mordor*” from a scene in the film adaption of J.R.R. Tolkien's Lord of the Rings) (Figure 7).

This motive of the following joke is also quite popular among twitter users: *“- Колян, мне кажется чай пахнет коноплей. – Нет. - Да пахнет, принюхайся. – Нет, Иван-чаем пахнет. - Чо хочешь сказать, что вчера Иван-чай курили”* (“- Kolyan, it seems to me that tea smells like hemp. - Not. - Smells, sniff it. - No, Ivan-chaj smells. - What do you want to say, that yesterday we smoked Ivan-chaj?”) (Twitter, user @ na-a, 1-Sep-19).

**Figure 8:**
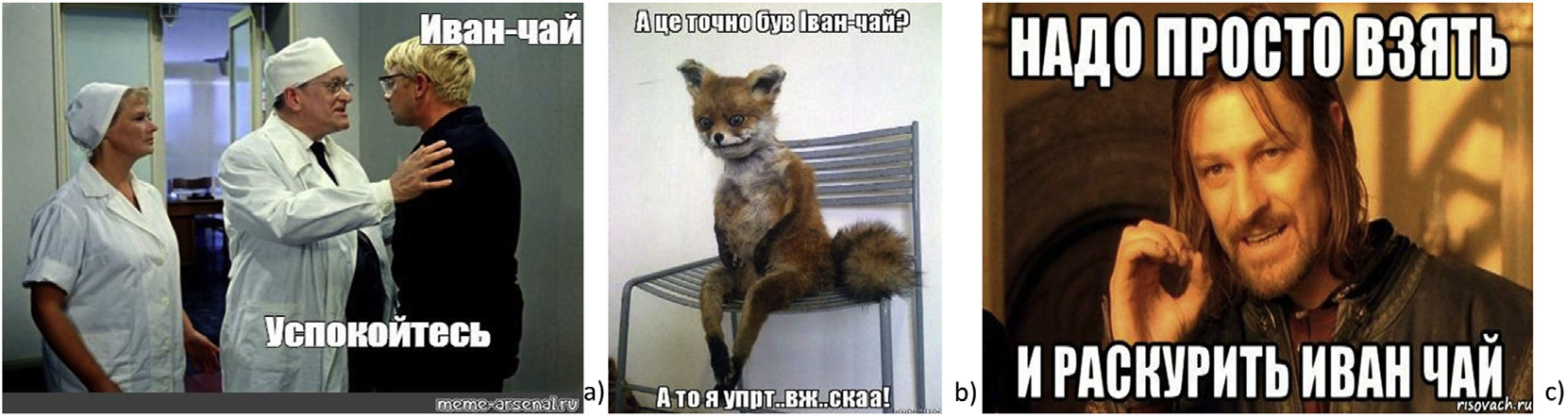
a) *“Ivan-tea! Make your mind easy!”* (meme-arsenal.ru); b) *“Was it definitely Ivan-tea? – I got stoned!”* (memesmix.net); c) *“You just need to smoke Ivan-tea”* (risovach.ru).

**Figure 9:**
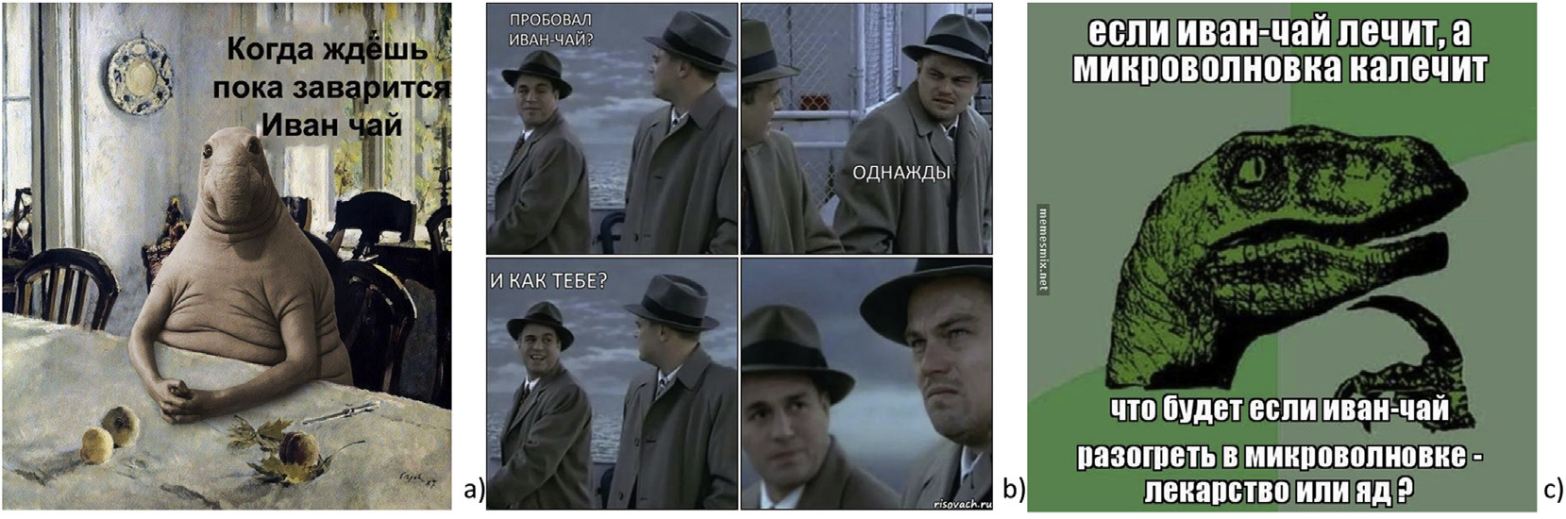
a) *“When you are waiting for Ivan-tea to brew”* (vk.com); b) *“Have you tasted Ivan-tea? - Once. - How did you like it? - …”* (risovach.ru); c) *“If Ivan-chaj heals, and microwaves disable, what will happen if Ivan-chaj is heated in a microwave - drug or poison?”* (memesmix.net).

Local motives are successfully combined with modern global forms and elements (Figure 8). The popular Snorp meme was photoshopped into the famous Russian work of art “Girl with peaches”; in a scene from Martin Scorsese's movie “Shutter Island”, the taste of Ivan-chaj was questioned; the Philosoraptor dinosaur (part of a series on advice animals) deeply immersed in metaphysical inquiries) (see Figure 9).

**Figure 10:**
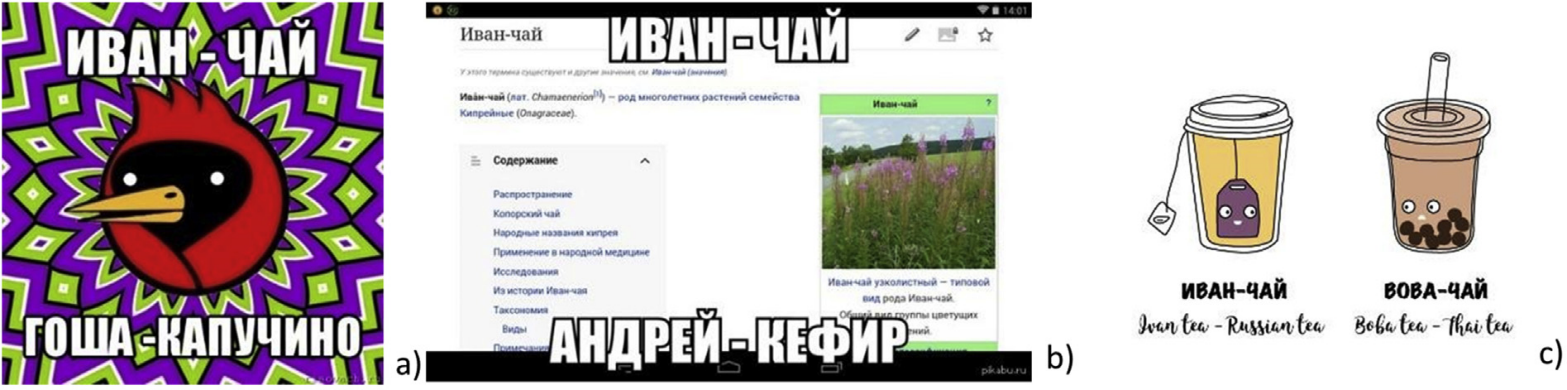
a) “*Ivan-tea. Gosha – cappuccino*” (risovach.ru); b) “*Ivan – tea. Andrei – kefir*” (pikabu.ru); c) “*Ivan-tea. Ivan tea – Russian tea. Vova-tea. Boba tea – Thai tea*” (vk.com).

We can also note a group of linguistic jokes about Ivan-chaj using wordplay, such as homography (words identically spelled in different languages), which involves exploiting the ambiguous meanings of words for humorous or rhetorical effect (Figure 10): “*пью иван-чай в надежде, что стану сегодня николаем”* (“I drink Ivan chaj in the hope that I will become Nicholas today”) (Twitter, user @T-na, 11-Jul-16); “*иван-чай, катя-водка”* (ivan-tea, katya-vodka) (Twitter, user @wh-y, 1-Nov-16); “*Иван чай ака джонни ти*” (“Ivan tea aka Johnny tea”) (Twitter, user @R–S, 18-Jan-15). Also, “*Что-то у них много Джеков: джек-пот, джек юнион, блэк джек, джек рассел терьер, джек потрошитель. А у нас только Иван-чай*” (“Somehow they have too many Jacks: jackpot, Union jack, Blackjack, Jack Russell terrier, Jack the Ripper. And we have only Ivan chaj”) (Twitter, user @h-om, 19-Mar-14).

#### 3.2.4 Virality of the grand narrative of “Russian tea” and its economic impact

Winner of the Nobel Prize in Economics Robert J. Shiller defined the term “viral narrative” as “a simple story or easily expressed explanation of events that many people want to bring up in conversation, on the news or on social media because it can be used to stimulate the concerns or emotions of others and/or because it appears to advance self-interest”. Shiller (2017) suggested that it is hard to predict which narratives, their elements or their mutations suddenly become popular or “go viral”, but once they do so, they “spread far, even worldwide, with economic impact”. He also stressed that “narratives are major vectors of rapid change in culture, in zeigeist, and ultimately in economic behavior” if the story can establish a “reference point, which has influence on decisions”. The “reference point” of Ivan-Chaj was certainly established, as by 2018 nine patents concerning willowherb had been issued; four of them involve tea production (Rospatent, 2018). There are more than 70 producers of Ivan-chaj in the Russian Federation, and the business was profitable (Ivan-chaj, 2018).

The first documented Ivan-chai festival (*Праздник Иван-чая*) was celebrated in Nizhny Novgorod Oblast in 2010, and it subsequently became a yearly event claiming to be a revival of traditional holiday celebrations (Il'inich, 2011). Now, numerous Ivan-chaj festivals are organized throughout the Russian Federation which have a similar structure and are combined with fairs. The key events of Ivan-chaj holidays include picking the plant and fermentation workshops and demonstrations. At these events, a Russian tea ceremony with Ivan-chaj has recently been gaining popularity (Festival, 2018). The holiday usually is scheduled on the Ivan Kupala (St. John's night) (from 6 to 7 July in Russia), which is correlated with the legendary origin of the plant in modern Internet narratives (Recept, 2013). According to popular belief, it is the time of the summer solstice – the best time for all plant gathering including willowherb. The Ivan-chaj holiday organizers are usually private individuals – representatives of agro-tourism, ecovillages and other cultural institutions. There are suggestions to include Ivan-chaj on the list of local dishes within the framework of gastronomic tourism, for example, in Yamal (Chebakova, 2017).

In 2015, Ivan-chaj seemed to have been promised more official support by the state in the Russian Federation. On the wave of import substitution, the Public Chamber of the Russian Federation conducted public hearings on the issue: “Developing a legislative framework for the development of the Ivan-tea industry in the Russian Federation and the support of domestic Ivan-chaj producers”. The members believed it necessary to develop a National Standard for producing Ivan-chaj products from willowherb, to create several different kinds of Ivan-chaj, and even to established a professional holiday of Russian herbalists (Ivan-chaj Day), as well as to recommend the government to consider reducing tea imports and finding ways to promote Ivan-chaj products in foreign markets (Recommendations, 2015). However, “The National Union of Producers of “Russian Tea”, which was created in 2015 and organized these hearings, quickly collapsed, they did not even have time to register it (Ivan-chaj, 2018).

The growing cohort of Ivan-chaj producers in the Russian Federation tends to abstain from pseudoscience or even taking a critical stance and investing their time in dispelling the myths created by their predecessors (see, for example, an exhaustive explanation in (Vovnij, 2015)). Nevertheless, the value of Ivan-chaj itself is not questioned as it is still claimed to have proven quality and health-improving abilities. The latest major appearance of Ivan-chaj on Russian TV happened in 2017 (Eda, 2017). The stance has changed from myth-propagating to robust ‘science’: the viewers learned that fermentation technology was brought from China. The episode was concluded by tea tasting and dégustation of sophisticated dishes made with willowherb leaves and flowers. The matter of zero cost is less and less discussed in the media for obvious reasons, even though this is an important issue for a majority of the audience.

**Figure 11:**
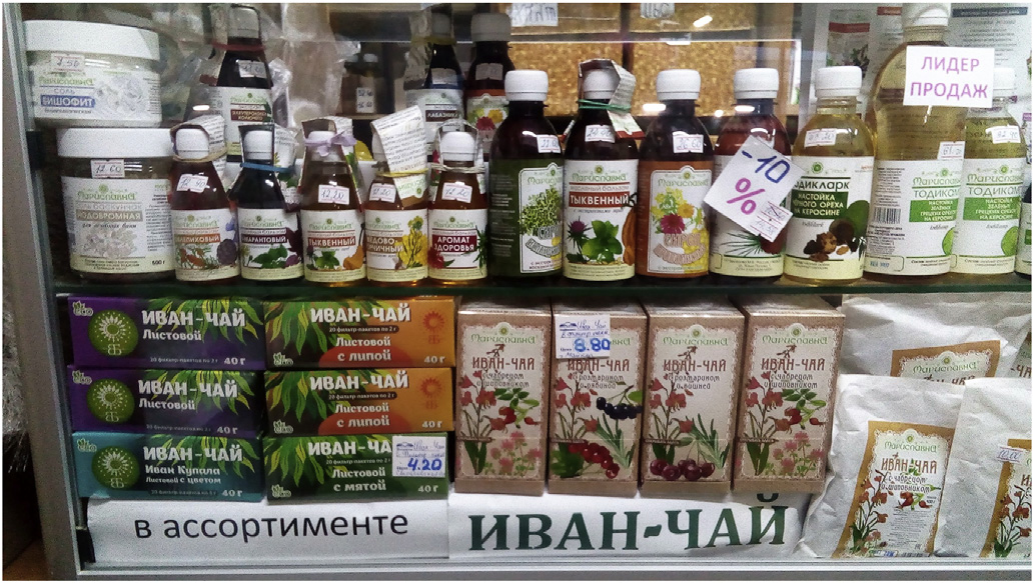
One of many examples of the highlighting of Ivan-chaj in local markets. Minsk, Kamaroŭski Market. February 2019. Photo by Julia Prakofjewa.

The fake history initially supporting the grand narrative of Ivan-chaj was also backed by food conspiracy narratives (cf. the narrative about yeast in bread (Kormina, 2016)) thanks to their presence in a majority of media outlets (newspapers, TV, social media). Together they could play a promoting role in consumer groups of various backgrounds, from younger educated urban dwellers to older inhabitants of rural areas. At the same time, Ivan-chaj sold in shops and local markets cannot be called cheap (Figure 11); for example, in a Belarusian market a pack of tea is sold for ca 4–10 Belarusian rubles, which is very expensive for people earning the minimum monthly salary of 325 rubles (as of 1 January 2019), a common salary in the countryside. Ivan-chaj is often presented as elite and a bioproduct, accessible only to wealthier individuals. However, there also seems to be some signs of over-production of Ivan-chaj, as, for example, a pack of it was offered as the final bonus to kitchenware sold on a TV shop commercial broadcast on the RenTV Baltic Channel, 11 July 2019.

Ivan-chaj has also recently been introduced as a regional food culture brand in the area around Lake Peipsi, the historic site of Russian Old Believers in Estonia. In 2015–2016, a village society in Lohusuu started to advertise itself to tourists through Ivan-chaj culture, offering trainings and presentations (Kiviselg, 2016) and publishing a leaflet (Tumanova, 2016), a regional recipe book, including instruction on producing Ivan-chaj (Tumanova and Oolberg, 2017), and an exhibition of regional food culture (including Ivan-chaj) in the Estonian National Museum (Laasik, 2017a, 2017b); supported by media publications (Rebane, 2017). However, the in-depth inventory of culinary traditions of Old Believers in Estonia did not mention the use of Ivan-chaj and the local plants were perceived mainly as taste additives to black tea (Kuvaitseva, 2010).

Mati Rebane, the natural therapist, translated legends about Ivan-chaj into Estonian and then published them on his website (Rebane, 2016). In a few months, these stories were published on the homepage of one of Estonia's most popular natural and ecological product distributors, looduspere.ee, despite the fact that this online store had already been selling Ivan-chaj with various additives for almost a year. The success of Ivan-chaj in Estonia has been supported by the long time promotion of its use against prostatitis, well publicized by popular herbalists and official medicine (Sõukand et al., 2020). By the end of 2018, several local small-scale companies produced, sold and introduced Ivan-chaj, supported by agricultural, entrepreneurship and regional programs in Estonia.

There are also numerous producers of Ivan-chaj in Belarus, Ukraine, Latvia, Lithuania, and other countries. It should be noted, though, that the Ivan-chaj holiday has not yet spread beyond the Russian Federation.

## 4 Conclusions

Following on from the idea of Shiller and our field results, we suggest that the wider a base the narrative has, the more people it addresses, and the greater chance it has to go viral and cause an economic impact. An overall positive image of Ivan-chaj was presented through the sum of all the storylines and other means of modern marketing formed into a general narrative, changing the way it was perceived. The diversity of narrative lines and their distribution created a situation where there was literally “something important” for everyone, and this most likely engaged a critical mass of people. Even the criticism of memes and jokes seems not to be able to ruin the positive image of Ivan-chaj. Currently, in the Russian Federation Ivan-chaj is drifting toward becoming a national culinary practice in which the taste, properties and relation to identity are regarded as the most important features of the drink. It also entered other countries through narratives on its medicinal usefulness and cultural importance, adding some more colorful narrative lines, best understood in the context of a given country. Plausible narratives were easily adapted for the potential audience and quickly spread by media. Having appeared in Russia, mostly as a Russian cultural marker, the narrative went viral and spread beyond its borders where neighbors tried in turn to embrace Ivan-chaj as their own cultural marker by proclaiming it a local tradition. Indeed, narratives regarding Ivan-chaj spread easily in countries sharing some linguistic, historical and/or cultural elements with Russia (via the nexus of the Soviet Union).

While the influence of the Internet and social media on the spread of invented tradition is not easily detectable and results may often not be univocally interpretable, we recommend future research in this direction in order to understand the differences between claimed and factual practice and the mechanisms of information transmission.

## Declarations

### Author contribution statement

Julia Prakofjewa, Raivo Kalle, Renata Sõukand: Conceived and designed the experiments; Performed the experiments; Analyzed and interpreted the data; Wrote the paper.

Olga Belichenko, Valeria B. Kolosova: Performed the experiments; Conceived and designed the experiments; Wrote the paper.

### Funding statement

This work was supported by the European Research Council (10.13039/100010663ERC) under the European Union's Horizon 2020 research and innovation programme (grant agreement No 714874).

### Competing interest statement

The authors declare no conflict of interest.

### Additional information

No additional information is available for this paper.
